# ECMO in Cardiogenic Shock: Time Course of Blood Biomarkers and Associated Mortality

**DOI:** 10.3390/diagnostics12122963

**Published:** 2022-11-26

**Authors:** Sasa Rajsic, Robert Breitkopf, Ulvi Cenk Oezpeker, Benedikt Treml

**Affiliations:** 1 Department of Anesthesiology and Intensive Care Medicine, Medical University Innsbruck, 6020 Innsbruck, Austria; 2 Department of Cardiac Surgery, Medical University Innsbruck, 6020 Innsbruck, Austria

**Keywords:** extracorporeal membrane oxygenation, ECMO, va-ECMO, hyperinflammation, inflammation, procalcitonin, C-reactive protein, mortality, outcomes, complications, adverse events

## Abstract

Background: Veno-arterial extracorporeal membrane oxygenation (va-ECMO) is a temporary life support for severe cardiogenic shock, gaining time for organ recovery, permanent assistance, or transplantation. In this work, we aimed to investigate the trends of blood biomarkers over the period of ECMO support and their role in patient outcome. Methods: This retrospective study comprised patients receiving va-ECMO support over the period of 14 years at a tertiary university center. Results: Of 435 patients, 62% (268/435) survived to discharge, and the most frequent adverse event was hemorrhage (46%), followed by thrombosis (25%). Deceased patients had increased blood levels of C-reactive protein, procalcitonin, and white blood cells during the whole observation period, with higher peaks compared with survivors. The multivariable model identified hemorrhage (HR 1.73, *p* = 0.005) and higher levels of procalcitonin (HR 1.01, *p* = 0.001) as independent risk factors for death. Conclusions: In our population of critically ill patients receiving va-ECMO support, deceased patients had increased inflammatory biomarkers during the whole observation period. Patients having higher values of procalcitonin and experiencing bleeding events showed an increased risk for mortality. Further studies focusing on inflammation in ECMO patients, clarifying its role in patient outcome and potential therapeutic interventions, are warranted.

## 1. Introduction

Extracorporeal membrane oxygenation (ECMO) is a temporary life support for cardiac or respiratory failure, gaining time for organ recovery, permanent assist, or transplantation. The veno-arterial (va-ECMO) configuration is used in a severe cardiogenic shock when conventional therapy fails, and the risk of mortality reaches 80% [1].

Multiple studies tried to identify predictors and risk factors for adverse events and mortality during ECMO support; however, inconsistent results indicate a need for further research [2,3,4,5,6,7]. It is well-established that hemorrhage has a strong impact on mortality [8,9], but studies failed to identify unique risk factors for bleeding [10,11,12,13,14,15]. Recent studies showed that patients with thrombosis had lower mortality, questioning therapeutic anticoagulation during ECMO support [12]. Previous research focused on the comparison of different laboratory parameters and complications occurrence on a certain day of support, missing the complete ECMO period [10,13,15,16]. However, information on the trend of biomarkers during ECMO support and its role in patient outcome is still missing.

In this work, we aimed to present and investigate the course of blood biomarkers over the period of ECMO support and their role in patient outcomes. Moreover, we provide a summary of the demographic and clinical characteristics of patients with refractory cardiogenic shock undergoing va-ECMO support.

## 2. Materials and Methods

### 2.1. Study Population and Data Acquisition

We reviewed medical charts of all patients receiving ECMO support admitted to intensive care units of the Department of Anesthesiology and Intensive Care Medicine of the Medical University of Innsbruck, Austria. The observation period included 14 years, from January 2008 to December 2021. Exclusion criteria were ECMO indication other than cardiogenic shock, patients having multiple ECMO initiations, duration shorter than 24 h, patients younger than 15 years, or incomplete data.

We obtained (a) patient socio-demographic data; (b) basic disease and indication for extracorporeal life support; (c) the Sequential Organ Failure Assessment (SOFA) and simplified the acute physiology III (SAPS III) score on ICU admission, presence of mechanical cardiopulmonary reanimation before or during ECMO initiation and duration of ECMO support; (d) data on complications, with date and cause of death; and (e) the use of anticoagulation. Within the laboratory parameters, we collected data on (a) coagulation status, including platelets count (g/L), fibrinogen (modified Clauss method, mg/dL), rotational thromboelastometry (ROTEM), and antithrombin (%) and (b) hemoglobin (g/L), erythrocytes (T/L), leucocytes count (g/L), C-reactive protein (CRP, mg/dL), and procalcitonin (PCT, µg/L).

Laboratory data were collected within 24 h before ECMO support initiation, daily during the observation period, and finally on days 3 and 10 after ECMO-support termination. The observation period was limited to a maximum of 14 days. This threshold is based on the median duration of ECMO support in our patient sample and the fact that more than 95% of patients needed ECMO support for less than two weeks.

Two authors (SR, BT) independently reviewed each electronic medical chart and extracted the socio-demographic and clinical data. This work was approved by the Ethics Committee of the Medical University of Innsbruck, Austria. Finally, the manuscript was prepared and revised according to the reporting of observational studies in the Epidemiology (STROBE) Statement—Checklist of items (Supplementary Table S1) [17].

### 2.2. ECMO-Support Management and Anticoagulation

The indication for ECMO support initiation was contrived by mutual judgment of a critical care specialist, a cardiac anesthesiologist, and a cardiac surgeon. At our institution, we utilize an ECMO system with a centrifugal pump, a hollow-fiber oxygenator, and a heparin-coated ECMO circuit. Refractory cardiogenic shock was defined as the need for vasopressors in order to maintain a systolic blood pressure above 90 mmHg or, in a case of a persistent low cardiac index (under 2.2 L/min/m^2^), combined with signs of end-organ dysfunction despite the use of all therapeutic options, such as inotropes and vasopressors [1,18].

The decision on blood- and coagulation-products substitution was made based on the clinician’s opinion and institutional standard operating procedures. In short, a minimum hemoglobin blood level of 8 g/dL is maintained, and bedside coagulation monitoring is utilized. However, we employ an individualized patient approach to tailor coagulation and bleeding management to the patient and the underlying disease.

Therapeutic anticoagulation was conducted according to the institutional standard operating procedure and based on the Anticoagulation Guideline provided by ELSO [19,20]. In general, we used unfractionated heparin as the first choice for anticoagulation (with a target activated partial thromboplastin time of 50–70 s, aPTT). Argatroban was used in case of inadequate anticoagulation or a suspected or proven heparin-induced thrombocytopenia type II. In case of a severe hemorrhagic diathesis, continuous anticoagulation was paused. Anticoagulation monitoring and adaptation was based on the activated clotting time (ACT), aPTT, anti-factor-Xa-assay activity, ROTEM^®^, or blood drug concentration of argatroban.

In the presence of cardiac function improvement signs (in echocardiographic evaluation) the ECMO-weaning protocol was initiated with a stepwise reduction of extracorporeal blood flow. A trial off was started following joint clinical judgment and a reduction of the ECMO blood flow under 30% of the total. Furthermore, the decision to withdraw ECMO support was made in case of unfavorable prognosis due to severe brain or irreversible heart damage or multiple organ failure without possibility for recovery.

### 2.3. Outcomes

The primary endpoint of our study was the association of the laboratory parameters course with in-hospital mortality. Secondary endpoints included the analysis of demographic and clinical characteristics, including the type and incidence of adverse events during ECMO.

Reported adverse events included hemorrhage, thrombosis, and sepsis. Data on thrombosis (localization and date of identification) were collected from the charts and radiology reports. The observation period included the ECMO-support period and 14 days after ECMO termination, as ultrasound or computed tomography may have been performed with delay and not necessarily during the ECMO support. Thrombosis was stratified into arterial and venous, depending on its localization.

We collected the data on bleeding events only during the ECMO support, and any hemorrhage thereafter was not considered as ECMO-related. Bleeding events were defined as minor or major, as by the ELSO definition [20].

The information on the date and cause of death were collected from the integrated medical documentation or autopsy report when available. Mortality in different periods was calculated based on the recorded date of death.

### 2.4. Statistical Analyses

Statistical analyses were performed using SPSS (version 28.0, released 2021, Armonk, NY, USA: IBM Corp.) and the R programming language (version 4.0.2, R Core Team 2020—free software for statistical computing and graphics: a language and environment for statistical computing; R Foundation for Statistical Computing, Vienna, Austria). The significance level of 0.05 was used. Depending on the data normality and type of variables, results are presented as mean (standard deviation), median (minimum and maximum), and frequency with percent. For parametric data, we used the independent samples t-test. For numeric and ordinal data with a non-normal distribution, we used the Mann–Whitney U test. We employed the chi-square test and Fisher’s exact test to analyze differences between nominal data. The univariate Cox regression analyses were employed to estimate the effect of potential risk factors on in-hospital mortality, and all significant variables were assessed in the multivariate model (*p* values under 0.05). We repeated multivariate models to explore the association of mortality and inflammatory biomarkers over time.

## 3. Results

### 3.1. Patient Characteristics

Within the observation period, 435 patients met the inclusion criteria and were included in the final analysis. The main ECMO indications were failure to wean from the cardiopulmonary bypass after heart valve surgery (158/435, 36%) and acute heart failure (121/435, 28%) (Table 1). The median SAPS III score and SOFA score were 62 (15–104) and 11 (1–21), respectively. Survivors experienced lower SOFA scores regarding the cardiovascular, neurological, and renal function compared to decedents.

The ICU length of stay was 18 (2–170) days. Finally, 62% (268/435) of patients survived to discharge and were discharged from hospital (Table 1).

The median ECMO-support duration was six days (2–22), in 71% (308/435) lasting shorter than seven days (Table 2). Anticoagulation of patients was primarily utilized with unfractionated heparin (312/435, 72%) and argatroban (71/435, 16%). Switching from unfractionated heparin to argatroban was recorded in 3% (12/435). Due to severe coagulopathy or life-threatening bleeding, 9% (38/435) of patients did not receive anticoagulation at all.

### 3.2. Laboratory Parameters during ECMO Support

We analyzed in total 19 laboratory parameters over the observed period and compared them between survivors and decedents. The graphical presentation revealed CRP, PCT, and white-blood-cell count (WBC) were higher in deceased patients compared to survivors.

**Figure 1:**
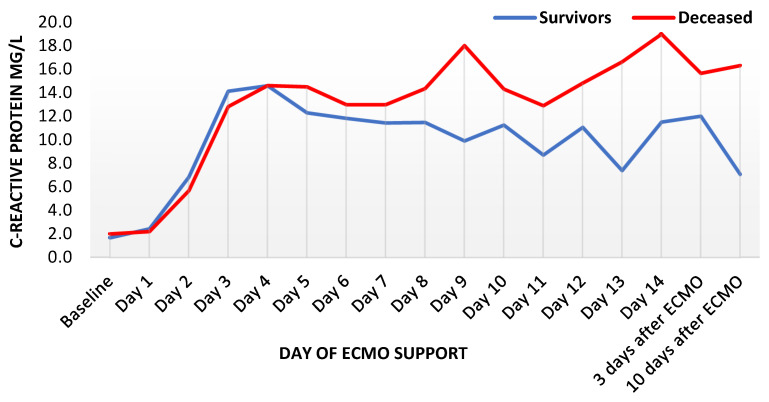
Trends of C-reactive protein levels during ECMO support, presented as median values (*n* = 435). ECMO: extracorporeal membrane oxygenation.

**Figure 2:**
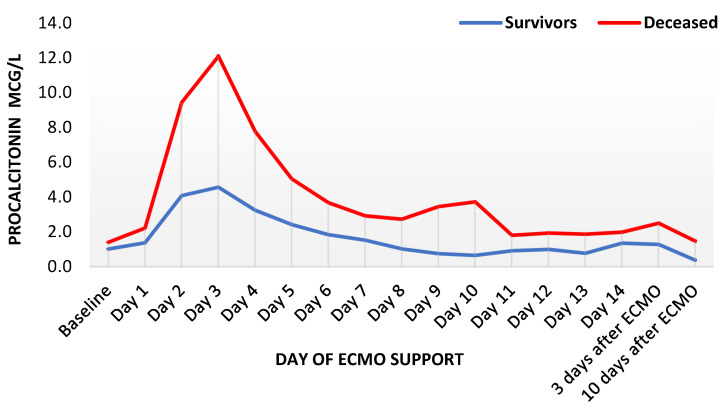
Trends of procalcitonin levels during ECMO support, presented as median values (*n* = 435). ECMO: extracorporeal membrane oxygenation.

**Figure 3:**
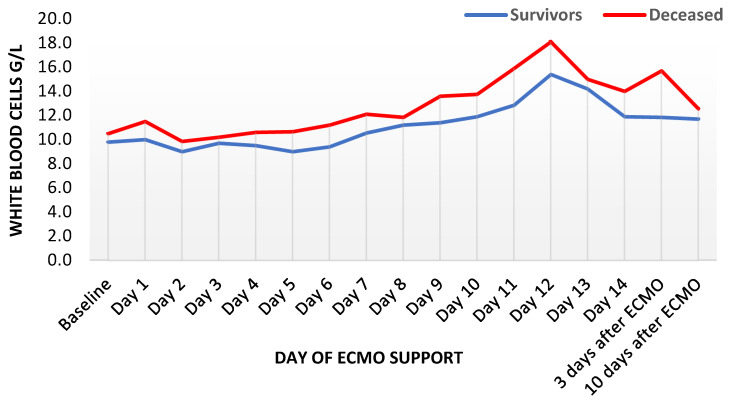
Trends of white-blood-cell count during ECMO support, presented as median values (*n* = 435). ECMO: extracorporeal membrane oxygenation.

C-reactive protein rose similarly fast in both groups until the third day of ECMO support, remaining increased for several days and distinctly higher than in survivors. Thereafter, a slow but steady decrease was observed in survivors until the end of the observation period. However, CRP remained increased in deceased patients (Figure 1). Procalcitonin reached its peak in both groups on the third day, with the maximum in decedents being nearly thrice as high as in survivors (median 4.6 mcg/l in survivors and 12.1 mcg/l in deceased patients, Figure 2). Thereafter, PCT decreased expeditiously for several days in both groups with a delayed flattening of the decrease. Moreover, PCT remained always higher in the deceased. Finally, WBC showed an unremarkable course during the first week, with a minor increase during the second week. Throughout the whole observation period, WBC remained constantly higher in deceased patients (Figure 3). Fibrinogen levels decreased by one third within one day and showed an increase after the second day, with a peak shortly after other inflammatory parameters reached their peaks (Supplementary Figure S1).

**Figure 4:**
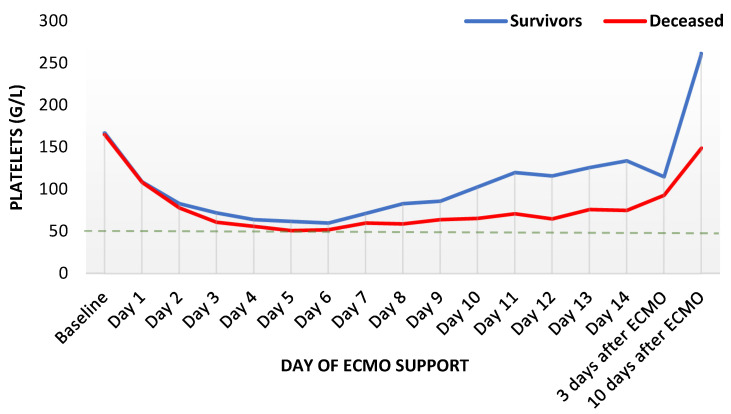
Trends of platelet count during ECMO support, presented as median values (*n* = 435). ECMO: extracorporeal membrane oxygenation.

Platelets decreased to one third of baseline values for several days, reaching the nadir on day six (Figure 4). Thereafter, platelets increased faster in survivors. However, both groups never reached baseline values again during the ECMO support.

Antithrombin showed a similar course with a distinct decrease until the third day and a slow recovery thereafter, being faster in survivors (Supplementary Figure S2). All patients demonstrated similar median values of hemoglobin (around 9 g/L) over the time course (Supplementary Figure S3).

### 3.3. Adverse Events during ECMO Support

The most frequent adverse event was hemorrhage (199/435, 46%), followed by thrombosis (110/435, 25%) and sepsis (80/435, 18%) (Table 2). Overall, in-hospital mortality was 38% (167/435) with the main causes of death being cardiac failure (68/435, 42%) and multiple-organ dysfunction syndrome (51/435, 31%) (Table 2).

Deceased patients experienced more often hemorrhage (93/167, 56%, *p* = 0.001), including a higher portion of major hemorrhagic events (59/167, 35%, *p* = 0.001). The overall incidence of thromboembolic events remained unchanged between both groups. However, deceased patients experienced more often arterial thrombosis (34/167, 20%, *p* = 0.018). Moreover, sepsis was observed in a quarter of deceased patients, being more frequent in this group of patients (40/167, *p* = 0.022).

Finally, multivariate Cox regression models identified higher values of PCT on day two, three, four, or five and any bleeding event during ECMO support to increase the hazard ratios for in-hospital mortality (Table 3, model with procalcitonin on day 3).

## 4. Discussion

This study represents the largest report investigating trends of commonly used blood biomarkers during ECMO support in cardiogenic shock. In our population of critically ill patients, deceased patients demonstrated higher levels of CRP, PCT, and WBC, with higher peaks compared to survivors and during the whole observation period. Regarding complications, ECMO treatment was more often complicated by hemorrhage and thrombosis, with an overall in-hospital mortality of 38%. Finally, higher values of PCT on the third day and bleeding events during ECMO support showed increased hazard ratios for mortality.

We observed a one-year mortality of 40%, which is rather low when compared to the available literature. A recent meta-analysis on mortality in cardiogenic shock in va-ECMO patients found an in-hospital mortality of 62% [9]. This discrepancy may be partly explained by undefined or different etiologies of cardiogenic shock and significant heterogeneity of included studies in the meta-analysis. Furthermore, we strive to initiate ECMO support only to patients after meticulous estimation of their respective prognosis.

Our multivariable model identified hemorrhage (HR 1.73, *p* = 0.005) during ECMO support as an independent risk factor for death, which is well-established in current literature [8,9]. Furthermore, this potentially lethal complication is independent of the type of ECMO configuration [4,6,21].

In regard to inflammatory markers, we observed a trend towards higher levels of acute phase proteins in deceased patients. The time courses of PCT, CRP, and WBC were comparable to data on the course of these blood parameters reported in literature [22,23,24]. Interestingly, a higher PCT was associated with an increased risk for death. This is in line with recent data from nearly 500 critically ill patients with sepsis, where a high level of PCT and an early increase on the first day have been shown as independent predictors of mortality [25]. A higher PCT in decedents could be partly explained through the initiation of an inflammatory response, which may be triggered (or at least favored) by the surgical trauma at the cannulation site and the continuous exposure of blood to the large artificial surface of the ECMO circuit [26]. From a physiologic view, increased levels of PCT may reflect a greater extent of inflammatory activation, as PCT has been shown to differentiate bacterial from non-bacterial infections [27,28]. However, during cardiogenic shock, gastrointestinal perfusion can be impeded. This can lead to bacterial translocation and a consequent increase of PCT [29,30]. Clearly, it is challenging to distinguish the extent of inflammation solely due to extracorporeal support or due to an underlying disease or even therapeutical and surgical interventions in patients on support [26,31]. Recently, an increased PCT has been shown to be non-discriminative between confirmed and suspected infection during ECMO in nearly 60 neonates and children [32]. Moreover, an increased PCT failed to estimate prognosis early in nearly 350 adult patients with va-ECMO support. Given the above, an increased PCT during ECMO may reflect some extent of inflammation despite its origin.

ECMO-related inflammation is less understood compared with inflammation related to cardiopulmonary bypass. Even if these two extracorporeal modalities are closely related and share several similarities, there are important distinctions that should be considered (e.g., duration of support, anticoagulation approach, use of protamine sulphate, temperature management, hemodilution, acute critical illness, potential multiple-organ dysfunction syndrome, etc.), which may further lead to different inflammatory responses [14,26].

The association of hyperinflammation with the occurrence of thrombosis, and a potentially reduced risk of bleeding, has already been described in literature [33,34]. Moreover, the methods of systemic inflammatory response syndrome after cardiopulmonary bypass minimization are being applied in daily clinical practice [35]. However, the evidence of a potential association of hyperinflammation and increased mortality in ECMO patients is still missing, as well as recommendations for its attenuation.

The question arises if any specific measures inhibiting such an unwanted exacerbation of the inflammatory response could be undertaken. In the case of cardiopulmonary bypass recommendations, including the application and further development of circuit bonding and leukodepletion, use of glucocorticoids, aprotinin, complement inhibitors, etc., exist [35]. However, in the case of va-ECMO, there is a paucity of evidence on the role of inflammation, originating mostly from retrospective or animal studies [26,33,36,37]. Further studies are warranted to clarify the role of inflammation in the outcome of ECMO patients and determine potential therapeutic interventions.

### Limitations

Certain limitations have to be kept in mind when interpreting our results. Given the retrospective nature of this work, selection bias cannot be excluded. Moreover, the information on the presence of an infection and its influence on the levels of biomarkers may have been limited given the missing possibility of a clinical assessment. The levels of fibrinogen, antithrombin, hemoglobin, and platelets were not evaluated in greater detail, as the measured levels could have been influenced by substitution of blood and coagulation products. Although this is one of the largest studies reporting on patients with ECMO in cardiogenic shock, we are not able to exclude a potential effect of missing data. This could hold especially true, as we were not able to analyze potential comorbidities or the need for continuous renal replacement therapy, which may influence the outcome [38,39]. However, we sought to provide an impression of the sickness of our patients using the SAPS III and SOFA score. Furthermore, it is complex to distinguish ECMO-related adverse events from potential complications of an underlying illness. Finally, we report on ECMO support outcomes over a period of 14 years. As meanwhile evidence on heart failure has evolved with new treatment options appearing, this may represent a potential limitation.

## 5. Conclusions

Here, we provide the largest report investigating the time course of the most common inflammatory biomarkers and their role in the outcome of patients with cardiogenic shock receiving ECMO support in a tertiary university center. Deceased patients demonstrated increased blood levels of PCT, CRP, and WBC during the whole observation period. Moreover, patients with higher levels of PCT and those experiencing hemorrhage had an increased risk for death. Further studies focusing on inflammation in ECMO patients, clarifying its role in patient outcome and potential therapeutic interventions, are warranted.

## Figures and Tables

**Table 1:** Demographic and clinical characteristics of patients with cardiogenic shock (*n* = 435).

Patient Characteristics		All Patients (*n* = 435)	Survivors (*n* = 268)	Deceased (*n* = 167)	*p*-Value	Missing Data (*n*/total)
Age (years)		60.8 ± 14.3	60.0 ± 14.2	62.1 ± 14.4	0.145	0/435
Male sex		303 (69.7)	190 (70.9)	113 (67.7)	0.520	0/435
Body mass index (kg/m^2^)		26.6 ± 4.6	26.4 ± 4.8	26.8 ± 4.4	0.438	3/435
SAPS III score		62 (15–104)	58 (15–99)	70 (31–104)	<0.001	1/435
SOFA score		11 (1–21)	11 (2–21)	11 (1–20)	0.021	1/435
SOFA respiratory		2 (0–4)	2 (0–4)	2 (0–4)	0.760	1/435
SOFA coagulation		1 (0–4)	1 (0–3)	1 (0–4)	0.464	1/435
SOFA liver		1 (0–4)	1 (0–3)	1 (0–4)	0.630	1/435
SOFA cardiovascular		4 (0–4)	4 (0–4), mean 3.5	4 (0–4), mean 3.8	<0.001	1/435
SOFA neurology		4 (0–4)	4 (0–4), mean 2.1	4 (0–4), mean 2.5	0.035	1/435
SOFA renal		1 (0–4)	1 (0–4), mean 0.9	1 (0–4), mean 1.1	0.045	1/435
CPR before ECMO initiation		118 (27.1)	62 (23.1)	56 (33.5)	0.020	0/435
Length of ICU stay (days)		18 (2–170)	21 (4–98)	11 (2–170)	<0.001	0/435
Cardiogenic shock etiology						0/435
No cardiotomy						
	Acute heart failure	121 (27.8)	63 (23.5)	58 (34.7)	0.009	
	Right heart failure	29 (6.7)	15 (5.6)	14 (8.4)		
Postcardiotomy						
	Coronary artery bypass surgery (CABG)	65 (14.9)	35 (13.1)	30 (18.0)		
	Heart valve surgery (HVS)	158 (36.3)	112 (41.8)	46 (27.5)		
	Combined (CABG and HVS, including aortic aneurysm)	38 (8.7)	25 (9.3)	13 (7.8)		
	Chronic heart failure	24 (5.5)	18 (6.7)	6 (3.6)		
Mortality-related outcomes						0/435
	Death during ECMO support	75 (17.2)	-	-		
	Death during ICU	154 (35.4)	-	-		
	Death within 90 days	166 (38.2)	-	-		
	Death within 365 days	173 (39.8)	-	-		
	Survived beyond one year	262 (60.2)	-	-		
Cause of death						4/167
	Cardiac	68 (41.7)	-	-		
	Respiratory	5 (3.1)	-	-		
	Brain death	19 (11.7)	-	-		
	Sepsis	20 (12.3)	-	-		
	MODS	51 (31.3)	-	-		

Data are presented as mean ± standard deviation, median (minimum–maximum), or number of patients (%). Abbreviations: CABG, coronary artery bypass surgery; CPR, cardiopulmonary resuscitation; ECMO, extracorporeal membrane oxygenation; HVS, heart valve surgery; ICU, intensive care unit; MODS, multiple-organ dysfunction syndrome; SAPS III, Simplified Acute Physiology Score III; SOFA, Sequential Organ Failure Assessment.

**Table 2:** ECMO-related characteristics and outcomes (*n* = 435).

Clinical Characteristics		All Patients (*n* = 435)	Survivors (*n* = 268)	Deceased (*n* = 167)	*p*-Value	Missing Data (*n*/total)
ECMO-related clinical course						0/435
	ECMO-support duration (days)	6 (2–22)	6 (2–20)	6 (2–22)	0.923	
	ECMO-support duration < 7 days	308 (70.8)	198 (73.9)	110 (65.9)	0.083	
Anticoagulation during ECMO support						0/435
	Unfractionated heparin	312 (71.7)	197 (73.5)	115 (68.9)	0.006	
	Argatroban	71 (16.3)	46 (17.2)	25 (15.0)		
	Epoprostenol	2 (0.5)	0 (0.0)	2 (1.2)		
	Switch from unfractionated heparin to argatroban	12 (2.8)	10 (3.7)	2 (1.2)		
	None	38 (8.7)	15 (5.6)	23 (13.8)		
Complications						
Hemorrhage		199 (45.7)	106 (39.6)	93 (55.7)	0.001	0/435
	Major hemorrhage	114 (26.2)	55 (20.5)	59 (35.2)	0.001	0/435
	Minor hemorrhage	85 (19.5)	51 (19.0)	34 (20.4)	0.804	0/435
Thromboembolic events		110 (25.3)	71 (26.5)	39 (23.4)	0.497	0/435
	Thrombosis venous	66 (15.2)	52 (19.4)	14 (8.4)	0.002	0/435
	Thrombosis arterial	65 (14.9)	31 (11.6)	34 (20.4)	0.018	0/435
Sepsis		80 (18.4)	40 (14.9)	40 (24.1)	0.022	0/435

Data presented as number of patients (%), mean ± standard deviation, or median (minimum–maximum). Abbreviations: ECMO, extracorporeal membrane oxygenation.

**Table 3:** Identification of risk factors for death: multivariate analysis (*n* = 435).

Variable	B-Coefficient	*p*-Value	HR	95% Confidence Interval	
				Lower	Upper
Procalcitonin on day three	0.008	0.001	1.01 ^a^	1.00	2.25
Resuscitation before ECMO	0.120	0.596	1.13	0.72	1.75
Surgical intervention	0.399	0.057	1.49	0.99	2.25
Hemorrhage	0.548	0.005	1.73	1.18	2.53
Arterial thrombosis	0.327	0.184	1.39	0.86	2.25

^a^ For every increase in one unit of measurement, hazard ratio increased by 1%. Abbreviations: ECMO, extracorporeal membrane oxygenation; CI, confidence interval; HR, hazard ratio.

## Data Availability

The datasets used and analyzed during the current study are made available from the corresponding author on reasonable request.

## Supplementary Materials

The following supporting information can be downloaded at https://www.mdpi.com/article/10.3390/diagnostics12122963/s1. Table S1, STROBE Statement—Checklist of items that should be included in reports of cohort studies; Figure S1, The course of fibrinogen over the observation period (*n* = 435); Figure S2, The course of antithrombin over the observation period (*n* = 435); Figure S3, The course of hemoglobin over the observation period (*n* = 435).
