# Supplementary material for: ECMO in Cardiogenic Shock: Time Course of Blood Biomarkers and Associated Mortality
Source: Diagnostics (Basel). 2022 Nov 26;12(12):2963. doi: 10.3390/diagnostics12122963 (PMC9776439; doi:10.3390/diagnostics12122963)

diagnostics

MDPI

ELECTRONIC SUPPLEMENTARY MATERIAL

ECMO in Cardiogenic Shock: Time Course of Blood Biomarkers and Associated Mortality

Sasa Rajsic [1], Robert Breitkopf [1], Ulvi Cenk Oezpeker [2] and Benedikt Treml [1,*]

[1] Department of Anesthesiology and Intensive Care Medicine, Medical University Innsbruck, 6020 Innsbruck, Austria
[2] Department of Cardiac Surgery, Medical University Innsbruck, 6020 Innsbruck, Austria
* Correspondence: benedikt.treml@i-med.ac.at; Tel.: +43-50504-22748

TABLE OF CONTENTS

Supplementary Table S1. STROBE Statement—Checklist of items that should be included in reports of cohort studies

Supplementary Figure S1. The course of fibrinogen over the observation period (n = 435)

Supplementary Figure S2. The course of antithrombin over the observation period (n = 435)

Supplementary Figure S3. The course of hemoglobin over the observation period (n = 435)

diagnostics

MDPI

Supplementary Table S1. STROBE Statement - Checklist of items that should be included in reports of cohort studies

No.	Item	Recommendation	Page
	Title and abstract		
1		(*a*) Indicate the study's design with a commonly used term in the title or the abstract.	1
		(*b*) Provide in the abstract an informative and balanced summary of what was done and what was found.	1
	Introduction		
2	Background/rationale	Explain the scientific background and rationale for the investigation being reported.	1
3	Objectives	State specific objectives, including any prespecified hypotheses.	1-2
	Methods		
4	Study design	Present key elements of study design early in the paper.	2-3
5	Setting	Describe the setting, locations, and relevant dates, including periods of recruitment, exposure, follow-up, and data collection.	2-3
6	Participants	(*a*) Give the eligibility criteria and the sources and methods of selection of participants. Describe methods of follow-up.	2-3
		(*b*) For matched studies, give matching criteria and number of exposed and unexposed.	
7	Variables	Clearly define all outcomes, exposures, predictors, potential confounders, and effect modifiers. Give diagnostic criteria, if applicable.	2-3
8*	Data sources/ measurement	For each variable of interest, give sources of data and details of methods of assessment (measurement). Describe comparability of assessment methods if there is more than one group.	2-3
9	Bias	Describe any efforts to address potential sources of bias.	2, 9
10	Study size	Explain how the study size was arrived at.	
11	Quantitative variables	Explain how quantitative variables were handled in the analyses. If applicable, describe which groupings were chosen and why.	3
12	Statistical methods	(*a*) Describe all statistical methods, including those used to control for confounding.	3
		(*b*) Describe any methods used to examine subgroups and interactions.	3
		(*c*) Explain how missing data were addressed.	3
		(*d*) If applicable, explain how loss to follow-up was addressed.	
		(*e*) Describe any sensitivity analyses.	
	Results		
13*	Participants	(a) Report numbers of individuals at each stage of study—e.g., numbers potentially eligible, examined for eligibility, confirmed eligible, included in the study, completing follow-up, and analysed.	3-8
		(b) Give reasons for non-participation at each stage.	3-4
		(c) Consider use of a flow diagram.	
14*	Descriptive data	(a) Give characteristics of study participants (e.g., demographic, clinical, social) and information on exposures and potential confounders.	3-8
		(b) Indicate number of participants with missing data for each variable of interest.	3-8
		(c) Summarize follow-up time (e.g., average and total amount).	2-3

15*	Outcome data	Report number of outcome events or summary measures over time.	3-8
16	Main results	(*a*) Give unadjusted estimates and, if applicable, confounder-adjusted estimates and their precision (e.g., 95% confidence interval). Make clear which confounders were adjusted for and why they were included.	3-8
		(*b*) Report category boundaries when continuous variables were categorized.	3-8
		(*c*) If relevant, consider translating estimates of relative risk into absolute risk for a meaningful time period.	
17	Other analyses	Report other analyses done—e.g., analyses of subgroups and interactions and sensitivity analyses.	3-8
	Discussion		
18	Key results	Summarize key results with reference to study objectives.	8-9
19	Limitations	Discuss limitations of the study, taking into account sources of potential bias or imprecision. Discuss both direction and magnitude of any potential bias.	9
20	Interpretation	Give a cautious overall interpretation of results considering objectives, limitations, multiplicity of analyses, results from similar studies, and other relevant evidence.	8-9
21	Generalizability	Discuss the generalizability (external validity) of the study results.	9
	Other information		
22	Funding	Give the source of funding and the role of the funders for the present study and, if applicable, for the original study on which the present article is based.	10

*Give information separately for exposed and unexposed groups.

diagnostics

MDPI

Supplementary Figure S1. The course of fibrinogen over the observation period (n = 435).

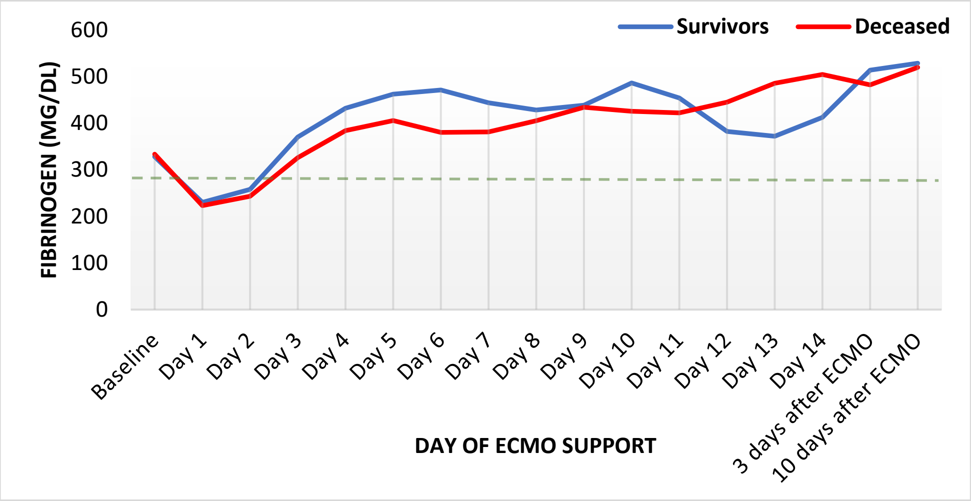

Supplementary Figure S2. The course of antithrombin over the observation period (n = 435).

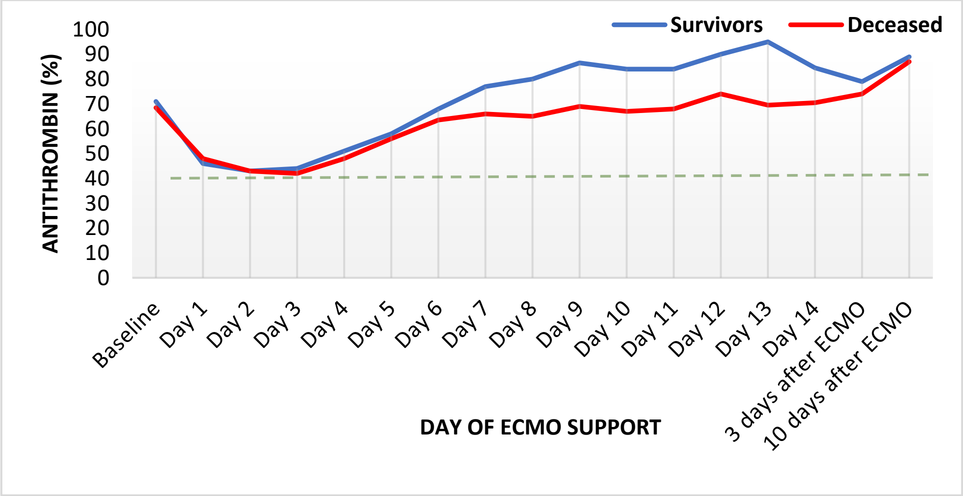

Supplementary Figure S3. The course of hemoglobin over the observation period (n = 435).

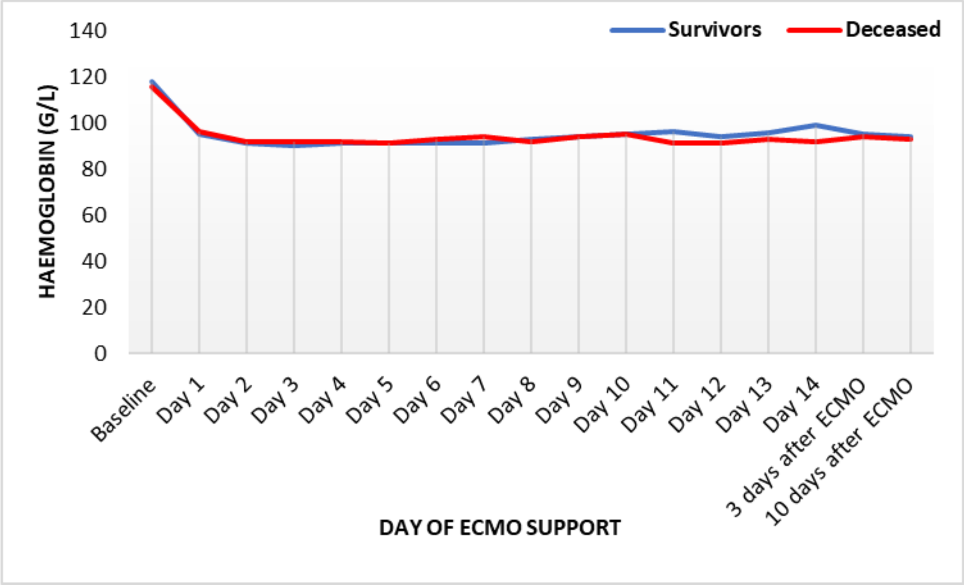

Supplement: Supplementary file 1 [file diagnostics-12-02963-s001.zip › diagnostics-2050921-supplementary.pdf]
